# EFFECTS OF SELF-PERCEPTIONS OF AGING ON COGNITIVE HEALTH: MEDIATING ROLE OF SOCIAL ISOLATION AND LONELINESS

**DOI:** 10.1093/geroni/igad104.1668

**Published:** 2023-12-21

**Authors:** Rita Hu, Lydia Li, Toni Antonucci, Ruth Dunkle, Jacqui Smith, Alexandra Crampton

**Affiliations:** University of Michigan, Ann Arbor, Michigan, United States; University of Michigan, Ann Arbor, Michigan, United States; University of Michigan, Ann Arbor, Michigan, United States; University of Michigan, Ann Arbor, Michigan, United States; University of Michigan, Ann Arbor, Michigan, United States; Marquette University, Milwaukee, Wisconsin, United States

## Abstract

Cognitive decline is one of the most common age-related stereotypes. The stereotype embodiment theory suggests that negative age stereotypes can be internalized through socialization and become negative self-perceptions of aging (SPA). However, the potential mediating role social relationships play between SPA and cognitive health remains unclear. Using three-wave data spanning eight years (2008/2010 – 2016/2018) from the Health and Retirement Study, this study aims to examine (1) SPA’s long-term effects on changes in cognitive health; and (2) the mediating role of social isolation and loneliness (N = 3,569, Mage = 72.30). SPA was measured by an 8-item scale. Cognitive health was measured by immediate and delayed word list recall. Social isolation was measured using network size, network diversity, frequency of contact with networks, and frequency of social participation. Loneliness was measured using the 11-item UCLA Loneliness scale. We controlled for baseline measures of the dependent variables and health-related and sociodemographic covariates. We found that negative SPA at baseline was associated with memory decline eight years later (b = -0.03, SE = 0.02, p < 0.05). The path analysis revealed that social isolation and loneliness were mediators between SPA and cognitive health, with social isolation accounting for 15% and loneliness accounting for another 32% of the total effects. The results suggest that social isolation and loneliness are pathways by which SPA exerts adverse effects on memory among older adults. Negative SPA should be a target for intervention. In addition, social relationship building could alleviate the impact of SPA on cognitive health.

